# Opinion: the neural basis of vestibular evoked myogenic potentials. The cVEMP is a specific indicator of saccular function

**DOI:** 10.3389/fneur.2025.1705995

**Published:** 2025-11-26

**Authors:** Jonas Bruun Kjærsgaard, Herman Kingma

**Affiliations:** 1 Department of Otolaryngology, Head & Neck Surgery and Audiology, Aalborg University Hospital, Aalborg, Denmark; 2 Department of Clinical Medicine, Aalborg Universitet, Aalborg, Denmark

**Keywords:** saccule, cervical vestibular evoked myogenic potential (cVEMP), origin of cVEMP, otolith function, translational medial research, otolith testing, saccular function

## Introduction

In a recent review Curthoys et al. (1) reviewed animal studies examining the responsivity of the vestibular system to sounds and stated that, beyond doubt, the cVEMP is a specific indicator of saccular function. However, in our opinion, the debate about the origin of the cVEMP is not closed. In our review (2), published 9 months earlier, we paid special attention to the issue that a normal cVEMP does NOT exclude loss of saccular function, especially that of the clinically most relevant sustained saccular system (detecting head tilt and linear accelerations). Furthermore, we draw a more conservative conclusion that substantial contributions of non-saccular vestibular end-organs to the cVEMP response cannot be ruled out (2). As this may leave researchers and clinicians in confusion, we want to share the key differences in the interpretation of the cited animal studies and the clinical relevance of cVEMP.

## Discussion

### Clinical relevance of cVEMP: is cVEMP really a test of saccular function?

One highly clinically relevant issue is not addressed in the review by Curthoys et al. (1) In contrast to what is suggested in the title of their review, it is a simplification to state that the cVEMP is a specific indicator of saccular function. Curthoys et al. (1) state that cVEMPs result predominantly from direct stimulation of striolar hair cells and irregular afferents, i.e., the transient otolith system, which is only a small part of the otolith system with a thin otolith membrane and small otoconia. Based on the transfer function of the otolith systems, high-frequency cVEMP stimuli hardly move the thick otolith membrane with larger otoconia (high membrane mass and stiffness) on top of the extrastriolar hair cells; in line with that, regular extrastriolar afferents have a low sensitivity to the high-frequency transient/vibratory stimuli used in VEMP testing (3–5). Therefore, cVEMP testing hardly—if at all—probes the function of the clinically very important sustained otolithic system, which is responsible for detecting head tilt and linear acceleration. Clinically, this distinction is crucial: cVEMP's results cannot be equated with preserved saccular function as suggested by Curthoys et al. (1), just as normal bone conduction does not ensure functional hearing in daily life. Hence, the presence of cVEMP is NOT a specific indicator of saccular function in toto; it might only reflect normal function of striolar hair cells and irregular afferents. This directly limits the clinical relevance of the cVEMP.

### Specificity of cVEMP: is the cVEMP predominantly reflecting the function of saccular hair cells? Are other hair cells in the labyrinth involved too?

Regarding the other discussion addressed in the review by Curthoys et al. (1) about the actual origin of the cVEMP: Is this response really only related to direct stimulation of saccular hair cells, or may other hair cells in the labyrinth contribute? We agree with Curthoys et al. that the later animal studies by the Bordeaux group [Didier, Cazal et al.] convincingly demonstrate the sound sensitivity of fibres coming from the saccule in the guinea pig model (1). The question is, however, to what extent the saccule may *selectively* be activated by sound. Curthoys et al. (1) specifically highlight a finding of Cazals et al. (6) where they, after semi-selective damaging the ampullae and the utricular maculae, were still able to elicit a non-cochlear electrophysiological response. The number of hair cells in the utricular macula and the ampullae was reduced by approximately half of that of the controls. The saccular hair cell count was reduced by one fourth (6). So, these experiments supported the relevance of saccular hair cell contributions, but do not exclude ampullar of utricular hair cell contributions. Furthermore, the responses were measured from the *round window niche* [(6), p. 612, 614], and ***not from the*** ***saccular nerve*, **in contrast to what Curthoys et al. state [(1), p. 3]. Therefore, the responses seen could still be composed of contributions from all still intact hair cells in sacculus, utriculus, and ampullae, and does not selectively show saccular responses. In conclusion, the experiments by Cazals et al. (6) are unable to rule out a substantial contribution by ampullar and utricular hair cells to the cVEMP response, which Curthoys et al. suggests (1). To our knowledge, there are no studies at hand that show that ablation of the sacculus invariably abolishes the cVEMP, which would be the real proof that the cVEMP is a crucial indicator of saccular hair cell activation. We are still awaiting such evidence to that stated by Welgampola and Carey (7).

We will now address three key aspects regarding the studies dealing with the origin of the cVEMP.

### Co-activation of the utricle and its projections to the SCM

Curthoys et al. (1) state that “if semicircular canal neurons are activated at clinical test frequencies and intensities, it detracts from the specificity of the cVEMP as a test of saccular function”. We agree, and that also holds for utricular coactivation, which was previously documented by both Curthoys et al. and Zhu et al., among others (8–12).

Curthoys et al. state that there is overwhelming evidence documenting that the utricle and saccule afferents have differential projections. To support this statement, they refer to the literature documenting differential projections from the saccule and utricle to the **extraocular** muscles (13). However, no evidence is provided that the saccular projection to the neck muscles is stronger than the utricular one. Instead, Curthoys et al. (1) state that the saccule has a strong projection to the neck muscles, while omitting that the equal may be stated for the utricle. This gives a false impression of the saccule having a stronger projection to the SCM than that of the utricle. This impression is not consistent with the available evidence, as documented by two studies by Kushiro et al. (14, 15) which investigated the projections from the otolithic organs to the neck muscles, including the SCM. In fact, in one of these studies, there is even some evidence suggesting that the utricular projection to the neck muscles could possibly be stronger than the saccular projection (2, 14). In the other study, Kushiro et al. uncovered that the afferents from both the saccule and utricle often terminate at the same second-order neurons, which then project to cervical motor units. From this, Kushiro et al. [(15), p.413] themselves conclude that the saccule and utricle may form structural neuronal common pathways to the neck muscles, hence the ipsilateral sternocleidomastoid muscle. Therefore, we have the opinion that both the saccule and utricle seem to utilize the same second-order neurons to drive the ipsilateral SCM response. Thereby, the saccular predominance theory (16) is still under debate, as it is unlikely that saccular input is a strict necessity for the elicitation of the ipsilateral SCM response to sound, because of the utricular projection. Welgampola, Carey, and Colebatch previously discussed the projections from the otolithic organs to the SCM in the context of cVEMP when the ‘saccular predominance theory' was first published, and contested its soundness (7, 17).

### The definition of activated afferents

There is evidence that all end-organs are activated by sound at intensities within limits of safe exposure (8, 10–12). However, this evidence has been put into question, in the review at hand, based on the use of phase-locked or probability firing as a measure of sensitivity (1). There are experimentally at least two different ways used in the relevant literature to determine responsiveness with quite different outcomes (8–12, 18). Curthoys et al. (9) have previously defended the choice of using rate-change as the definition of activation over phase-locking or probability of firing. Rate-change sensitivities may only be studied using sustained tones (9, 10), or long tone-bursts (18). These types of stimuli are not used for human cVEMP testing, where clicks or short tone-bursts are the standard. So, the extrapolation of rate-change sensitivities to the cVEMPs in humans can be questioned. Furthermore, using rate-change will not take into account activation of afferents firing highly synchronized to the stimulus (19), if their total rate of firing is not exceeded [(18), p.6064, Figure 7]. Indeed, Young et al. (10), using sustained tones, found that phase-locking thresholds were 10–30 dB lower than those of rate-change thresholds. Equally, McCue and Guinan showed that phase-locking to click stimuli results in considerably lower thresholds than using rate-change to long tone-burst stimuli (18). All these aspects result in a complex and different interpretation of the contribution of non-saccular afferents to cVEMP (8, 10–12). So, for us, it is not at all clear what the relative contribution of non-saccular afferents is to cVEMP in humans, and thereby how specific the saccular contribution can be said to be.

### The sound intensity used in the rat studies

Zhu et al. (8, 11) used high stimulus intensities in the rat studies, which, according to Curthoys et al. (1) do not allow extrapolation to the standard stimulation intensities used in humans. However, Zhu et al. (8, 11), when observing considerable activation of afferents from all five vestibular end-organs used an intensity up to 130 dB ***peak*** SPL, and not 130 dB SPL as incorrectly stated by Curthoys et al. (1). This is an important distinction (20). The ABR threshold to click in the rats used by Zhu et al. is 50 dB peak SPL (21), which, after adding the 80 dB sensational level, equals 130 dB peak SPL, just like Zhu et al. state (8, 11). Rosengren et al. (22), examining cVEMP responses in humans using 0.1 ms clicks of 138.7 dB peak SPL, report a crest factor of up to 33.4 dB between peak SPL and the A-weighted, 1 s integrated SPL. In fact, 130 dB peak SPL, depending on the integration time used, may be equivalent to or < 100 dB SPL. The first cVEMP studies in healthy people reported usage of 0.1 ms clicks delivered at 145 dB SPL (23, 24), which might have been 145 dB *peak* SPL, which in any case is much higher than the intensities used by the Zhu-group in rats. In standard clinical cVEMP practice, the maximum intensity used is 135–140 peak SPL (25). Thereby, the intensities used by the Zhu-group are similar to or even below the values used clinically, and extrapolation to humans is therefore not hindered, as Curthoys et al. suggest (1). The focus on this core issue is shifted away in the review of Curthoys et al. (1) by the description of behavioural threshold differences between the rat, guinea pig, and humans, and related flawed speculations of the physical sound intensity used by Zhu et al. (8, 11).

## Summary

In its current form, the review advocates for the continued use of cVEMP as a specific measure of saccular function. Yet this endorsement rests on a misrepresentation of the studies and findings by Cazals et al. (6), Uchino et al. (13–15), and Zhu et al. (8, 11), as demonstrated herein. Moreover, the appropriateness of using rate-change as a criterion for irregular afferent activation has been questioned, given that it necessitates a stimulus paradigm that is not applicable to cVEMP testing.

Finally, and most clinically relevant, is the recognition that **an intact VEMP does not imply normal macular function**. Clinicians should be particularly aware of this limitation when managing patients with vestibular disorders.
